# Impact of PET data driven respiratory motion correction and BSREM reconstruction of ^68^Ga-DOTATATE PET/CT for differentiating neuroendocrine tumors (NET) and intrapancreatic accessory spleens (IPAS)

**DOI:** 10.1038/s41598-020-80855-4

**Published:** 2021-01-26

**Authors:** Virginia Liberini, Fotis Kotasidis, Valerie Treyer, Michael Messerli, Erika Orita, Ivette Engel-Bicik, Alexander Siebenhüner, Martin W. Huellner

**Affiliations:** 1 Nuclear Medicine Unit, Department of Medical Sciences, University of Turin, Turin, Italy; 2 GE Healthcare, Waukesha, WI USA; 3 Department of Nuclear Medicine, University Hospital Zürich, University of Zürich, Rämistrasse 100, 8091 Zurich, Switzerland; 4 Department of Hematology and Medical Oncology, University Hospital Zürich, University of Zürich, Zurich, Switzerland; 5 ENETS CoE Training Fellowship Grant 2019, Berlin, Germany

**Keywords:** Cancer, Diagnostic markers, Endocrine system and metabolic diseases

## Abstract

To evaluate whether quantitative PET parameters of motion-corrected ^68^Ga-DOTATATE PET/CT can differentiate between intrapancreatic accessory spleens (IPAS) and pancreatic neuroendocrine tumor (pNET). A total of 498 consecutive patients with neuroendocrine tumors (NET) who underwent ^68^Ga-DOTATATE PET/CT between March 2017 and July 2019 were retrospectively analyzed. Subjects with accessory spleens (n = 43, thereof 7 IPAS) and pNET (n = 9) were included, resulting in a total of 45 scans. PET images were reconstructed using ordered-subsets expectation maximization (OSEM) and a fully convergent iterative image reconstruction algorithm with β-values of 1000 (BSREM_1000_). A data-driven gating (DDG) technique (MOTIONFREE, GE Healthcare) was applied to extract respiratory triggers and use them for PET motion correction within both reconstructions. PET parameters among different samples were compared using non-parametric tests. Receiver operating characteristics (ROC) analyzed the ability of PET parameters to differentiate IPAS and pNETs. SUVmax was able to distinguish pNET from accessory spleens and IPAs in BSREM_1000_ reconstructions (p < 0.05). This result was more reliable using DDG-based motion correction (p < 0.003) and was achieved in both OSEM and BSREM_1000_ reconstructions. For differentiating accessory spleens and pNETs with specificity 100%, the ROC analysis yielded an AUC of 0.742 (sensitivity 56%)/0.765 (sensitivity 56%)/0.846 (sensitivity 62%)/0.840 (sensitivity 63%) for SUVmax 36.7/41.9/36.9/41.7 in OSEM/BSREM_1000_/OSEM + DDG/BSREM_1000_ + DDG, respectively. BSREM_1000_ + DDG can accurately differentiate pNET from accessory spleen. Both BSREM_1000_ and DDG lead to a significant SUV increase compared to OSEM and non-motion-corrected data.

## Introduction

Accessory spleens are congenital foci of healthy splenic tissue that are separate from the main body of the spleen^1,2^. Accessory spleens are relatively common, mostly solitary and have no gender predilection. Their presence was reported to be as high as 10–30% in postmortem studies^3^ and in 45–65% of subjects after splenectomy^4^. On CT, accessory spleens are seen in approximately 15% of patients^5^. Accessory spleens are typically located in the splenic hilum (80%) and in the tail of the pancreas (17%), but are also found in other locations, such as the greater omentum, the splenic ligament, the small and large intestinal mesentery, the wall of the small intestine, the female annex and the scrotum^3,6^.

Intrapancreatic accessory spleens (IPAS) appear as a solid contrast-enhancing mass, usually smaller than 3 cm, located within the tail of the pancreas^7–9^. Owing to their morphology and contrast characteristics, they may be mistaken for pancreatic tumors, in particular neuroendocrine tumors^10^. Both CT and MR imaging have limited ability to discriminate pancreatic NET and IPAS because they typically share similar morphology and contrast enhancement characteristics. Hence, an accurate diagnosis may avoid unnecessary surgery or biopsy.

Accessory spleens can be diagnosed with Tc-99m-labelled colloids. These colloids are taken up into splenic tissue due to phagocytosis in the reticulum-endothelial cells and, therefore, can also identify functioning ectopic splenic tissue^11^. Tc-99m-labelled heat-damaged red blood cell scintigraphy (Tc-99m-HDRBC) also utilizes reticulum-endothelial cells that decompose damaged red blood cells^12,13^. However, the sensitivity of these exams is hampered by the small size of most IPAS and by the comparably low resolution of conventional scintigraphy, limiting a more widespread use of these techniques.

^68^Ga-labeled somatostatin analogue such as ^68^Ga-DOTA-TOC, ^68^Ga-DOTA-TATE and ^68^Ga-DOTA-NOC PET is the mainstay for the evaluation of the somatostatin receptor (SSTR) status of neuroendocrine neoplasms. The biodistribution of somatostatin analogs is characterized by a physiological uptake in several organs, including spleen^14^ and ectopic splenic tissue. Hence, IPAS may mimic pancreatic neuroendocrine tumors (pNET) and cause a false positive finding.

Several retrospective studies assessed radiological imaging characteristics of accessory spleens^15,16^. Specific reports on ^68^Ga-DOTA-peptide PET in the literature are limited to individual cases^17–19^.

The aim of our study was to evaluate whether quantitative parameters of ^68^Ga-DOTATATE PET/CT can differentiate pNET from accessory spleens, including IPAS. For this purpose, the impact of novel Bayesian penalized likelihood reconstruction and respiratory data-driven motion correction of PET on quantitation were investigated^20^.

## Materials and methods

### Patient selection

**Figure 1:**
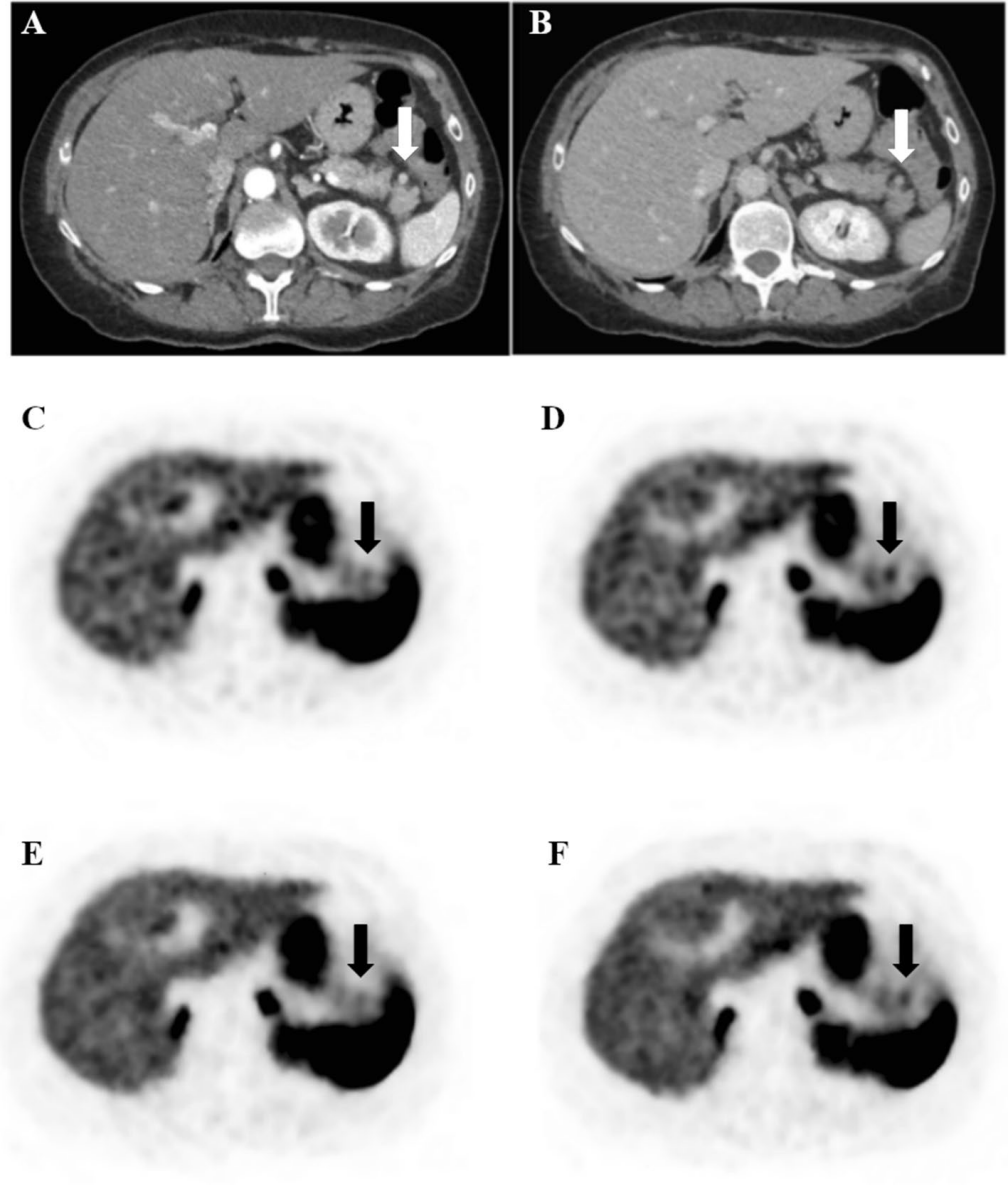
^68^Ga-DOTATOC PET/CT showing an intrapancreatic accessory spleen mimicking a pancreatic neuroendocrine tumor. Contrast-enhanced CT image in portal-venous phase showing the lesion in the pancreatic tail (**a**). Contrast-enhanced CT image in arterial phase showing the enhancing lesion in the pancreatic tail. The lesion is characterized by mild ^68^Ga-DOTATATE uptake, more evident in OSEM-DDG (**d**) and BSREM-DDG (**f**) images (SUVmax 8.6 and 7.6, respectively) compared with OSEM (**c**) and BSREM (**e**) images without DDG (SUVmax 6.4 and 6.2, respectively). The PET volume of the accessory spleen is smaller in OSEM-DDG and BSREM-DDG images (980 mm^3^ and 814 mm^3^, respectively) compared with OSEM and BSREM images without DDG (1400 mm^3^ and 1520 mm^3^, respectively).

**Figure 2:**
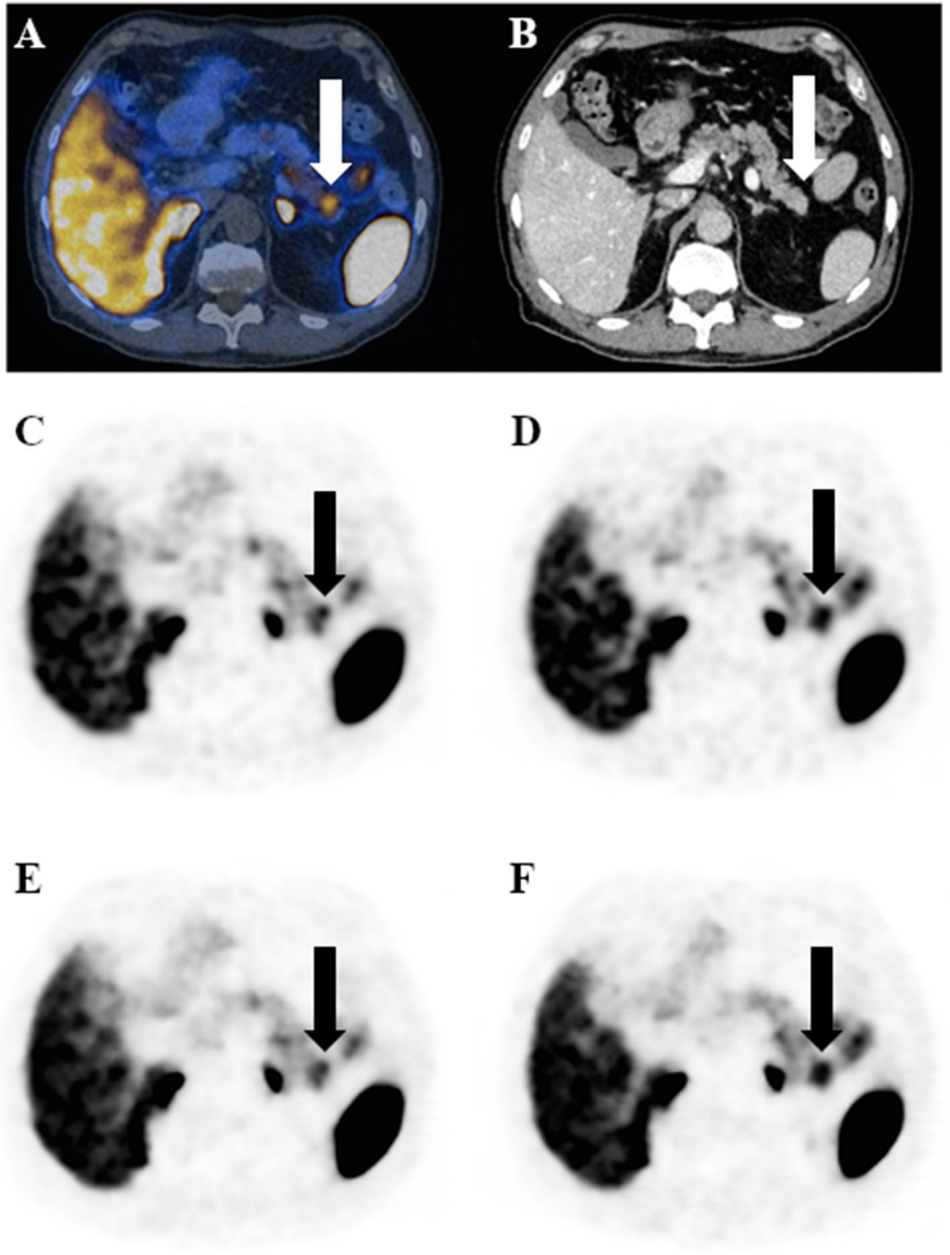
^68^Ga-DOTATATE PET/CT with a pancreatic neuroendocrine tumor seen on PET/CT image (**a**) and on contrast-enhanced CT image (**b**), confirmed by biopsy. Transaxial PET/CT images illustrate the impact of different reconstruction on morphology, volume and uptake of the neuroendocrine tumor. The lesion is better defined and yields higher SUVmax on OSEM-DDG (**d**) and BSREM-DDG (**f**) images (SUVmax 13.0 and 12.1, respectively) compared with OSEM (**c**) and BSREM (**e**) images (SUVmax 11.2 and 10.4, respectively). In this case, PET volume of the lesion is increased by DDG reconstruction (2880 mm^3^ for OSEM-DDG and 3170 mm^3^ for BSREM-DDG, respectively), because the severe motion blurring present in the conventional reconstruction (2650 mm^3^ for OSEM and 2920 mm^3^ for BSREM_,_ respectively) is compensated by DDG, yielding improved lesion conspicuity.

We retrospectively analyzed a cohort of 498 consecutive patients, who underwent a clinically indicated PET/CT with ^68^Ga-DOTATATE for staging/restaging neuroendocrine tumors (NET) at the University Hospital of Zurich between March 2017 and July 2019. Only patients with documented willingness to the use of their medical data for research were included in this retrospective, observational study. Our study was approved by the local ethics committee and was conducted in compliance with ICH-GCP rules and the Declaration of Helsinki. First, all PET/CT scans were reviewed by one nuclear medicine physician. All subjects with accessory spleens (28 patients and 38 list mode scans with a total of 43 accessory spleens) were included into the study. Intrapancreatic lesions were considered accessory spleens (IPAS) if lesions had been stable for at least two years, and in the absence of any abdominal neuroendocrine tumor history (n = 7). CT characteristics of lesions were reviewed by one double board-certified radiologist/nuclear medicine physician (Fig. 1). Subjects with pancreatic neuroendocrine tumors (pNET), either primary or metastatic, were included only if histopathology was available (7 patients and 9 list mode scans with a total of 9 pNET; 2 scans with 1 accessory spleen and 1 pNET already included in the accessory spleens cohort and 7 scans with 1 pNET) (Fig. 2).

### PET/CT acquisition

All patients underwent an exam on a PET/CT scanner with digital detector read-out technology equipped with silicon photomultipliers (SiPM) (GE Discovery Molecular Insights—DMI PET/CT, GE Healthcare, Waukesha, WI). In accordance with the EANM procedure guidelines for PET imaging, the injected tracer activity was 132.7 ± 20.9 MBq of ^68^Ga-DOTATATE (range 81–164 MBq).

After an uptake time of 60 min (range 52–79) and following CT acquisition for attenuation correction (from the vertex of the skull to the mid-thighs), PET data were acquired in time-of-flight (TOF) mode, covering the same anatomical region of the CT, with 2–3 min/bed position and 6–8 bed positions per patient (23% overlap). After that, following in-house rules for ^68^Ga-DOTATATE-PET, a contrast-enhanced CT (ceCT) scan was acquired (in arterial and venous phase) after intravenous injection of 70 mL of iodinated contrast medium (Iodixanol [VISIPAQUE 320]; GE Healthcare) in breath-hold, with tube voltage range 120–140 kV and automated dose modulation (range 60–440 mA/slice).

### Bayesian penalized likelihood (BPL) reconstruction algorithms

The use of absolute quantitative methods for PET/CT requires a fundamental standardization and harmonization^21,22^. The precision of quantitative PET metrics depends on the combination of several aspects, including homogeneity of reconstruction protocols and data analysis methods^23^. Today, ordered subset expectation maximization (OSEM) is the most widely used algorithm for clinical PET/CT image reconstruction^24^, as clinical standard-of-care method. However, it is known that the true SUV is consistently underestimated with OSEM owing to early iteration termination and subsequent under-convergence, which is traded-off against noise^25,26^. Bayesian penalized likelihood (BPL) reconstruction algorithms, such as block sequential regularized expectation maximization (BSREM—Q.Clear; GE Healthcare), are increasingly used in clinical routine. Indeed, BSREM increases the accuracy of lesion quantitation compared to OSEM by maximizing signal–to-noise ratio (SNR) while achieving almost full convergence^27–29^.

The global strength of the regularization in BSREM (β-value) can be choose and modulate according to the dataset of patients being studied, allowing for optimal reconstruction depending on the specific characteristics of the radiotracer (i.e.: images with low/high contrast or low/high noise, physiological distribution of the radiotracer). In this study, the β-value of 1000 was selected based on previous studies, analyzing the effect on image quality and considering both radionuclide properties and scanner characteristics^30–32^. In a recent study focusing on optimization of image reconstruction for ^68^Ga-PSMA-11, a similar β-value close to 900 provided an optimal tradeoff between signal and noise^33^.

### Respiratory tracking and correction

Respiratory motion may impact on quantification and qualitative evaluation of lesions in the chest and upper abdomen. Since most accessory spleens are found in this region, motion tracking and subsequent correction may alter quantitative indexes and hypothetical cut-off values between IPAS and pNETs. Traditionally, respiratory motion correction is performed using an external gating device positioned on the patient’s abdomen/chest and prescribing a priori the number and location of bed positions to correct for motion.

Unlike externally driven gating, which usually relies on infrared camera tracking of patient motion, data-driven gating methods use solely PET raw data in combination with dimensionality reduction techniques, in order to extract the respiratory signal. In our study, we used a commercially available DDG algorithm (MOTIONFREE, GE Healthcare), in combination with a motion correction algorithm (Q.STATIC, GE Healthcare) that utilizes the quiescent phase of the respiratory cycle [quiescent period gating (QPG)]^34–37^.

This technique utilizes a principal component analysis (PCA) to compute the spatiotemporal variation of list mode data. The algorithm provides a signal-to-noise measure of respiration-like frequencies within the data, denoted as *R-*value, that is configurable (*R-*value threshold). The determination of the *R-*value has a function of trigger: the *R-*value is measured at the end of base acquisition time for each bed position for which motion screening was prescribed and is used to make an on-the-fly decision whether motion has been detected. This then triggers the data acquisition to automatically be extended according to the prescribed acquisition time and data binning scheme used for motion correction (MC)^38^. In the default protocol, the quiescent phase of the respiratory cycle is set to 50% for motion correction, triggering an automatic data acquisition extension equaling to double the base acquisition time in order to preserve total count statistics. If data are screened retrospectively for motion, then using Q.STATIC with a 50% quiescent phase results in utilizing half the acquired data for MC without the ability to extend the acquisition prospectively. Furthermore, as the majority of data included in this work were analyzed retrospectively (42 scans for a total of 42 accessory spleens, 6 IPAS and 8 pNET) and not acquired prospectively with data acquisition extension, comparisons to non-MC data were performed using half the base scan time in order to have comparable datasets with similar count statistics. Scans acquired prospectively (3 scans for a total of 1 accessory spleen, 1 IPA and 1 pNET) was not included in this sub-cohort analysis to avoid bias in data analysis. The *R-*value for triggering motion correction used for this study was *R* = 10.0^37^*.*

### Image reconstruction

PET image datasets were reconstructed with different settings (all with a 256 × 256 pixel matrix):

1. OSEM: 3 iterations, 16 subsets, FWHMI of 6.3 mm, 1:4 Z-axis filter and 6.4 mm Gaussian filter with both time-of-flight (TOF) and point spread function (PSF) modelling (OSEM_PSF_; VUE Point FX with SharpIR, GE Healthcare).
2. BSREM (Q.Clear, GE Healthcare) with both TOF and PSF and a β-value of 1000.

For respiratory motion correction, we used the DDG algorithm (MOTIONFREE, GE Healthcare) in combination with a motion correction algorithm (Q.STATIC, GE Healthcare), using a phase offset of 30% and a phase window width of 50%, which are the default parameters supplied by the vendor:

1. BSREM_1000_-DDG with *R* = 10.0 + Q.STATIC;
2. OSEM-DDG with *R* = 10.0 + Q.STATIC.

### Quantitative imaging analysis

Quantitative analysis was performed by one reader and PET images were segmented using a General Electric AW workstation running PET VCAR software (GE Healthcare, Waukesha, WI, USA). The following indices were recorded for each lesion:

1. location,
2. maximum diameter in mm, measured on CT,
3. volume in mm^3^, measured both on CT and on PET (PET volume),
4. maximum standard uptake value (SUVmax) and mean standard uptake value (SUVmean).

The volume was measured on CT data with an automatic contouring tool, while on PET was calculated using a volume of interest (VOI) including the whole lesion volume, outlined with a 3D semi-automatic contouring tool, and applying a threshold set at 41% of SUVmax^21^. SUVmax and SUVmean were calculated from the same VOIs. Thereby the VOI was automatically propagated and adjusted to cover the lesion volume in all different reconstruction sets.

For all patients, a VOI of the spleen was also outlined on PET data and its volume measured in mm^3^ along with SUVmax and SUVmean. Furthermore, a reference liver ROI was used by drawing a 3 cm^3^ VOI in the right lobe of the liver (parenchymal organ background).

### Statistical analyses

All statistical analyses were performed using SPSS version 25.0 (IBM Corporation, Armonk, NY, USA^39^) and Minitab version 19.0 (Minitab Inc., State College, PA, USA^40^). Categorical variables are expressed as proportions, and continuous variables are presented as mean ± standard deviation (sd) or median (range), depending on the distribution of values. The Pearson correlation coefficient was used to assess the relationship between SUVmax and diameter (mm) of the accessory spleens. A linear regression equation of diameter over SUVmax value was calculated for all accessory spleens. Quantitative PET data (SUVmax and SUVmean) of the three samples (all accessory spleens, IPAS and pNETs) were compared in each reconstruction using Mann–Whitney U-test. *P*-values were calculated for IPAS versus all accessory spleens, IPAS versus pNET, and pNET versus all accessory spleens.

Parameters (SUVmax, SUVmean and volume) of each sample (all accessory spleens, IPAS, pNETs, and all lesions) were compared among different reconstructions using Wilcoxon signed ranks test. The different reconstructions were compared as follows:

- BSREM_1000_ versus OSEM;
- BSREM_1000_-DDG with *R* = 10.0 + Q.STATIC versus BSREM_1000_ half time/bed position (BSREM_1000_ 1/2);
- OSEM-DDG with *R* = 10.0 + Q.STATIC versus OSEM half time/bed position (OSEM 1/2);
- BSREM_1000_-DDG with *R* = 10.0 + Q.STATIC versus OSEM-DDG with *R* = 10.0 + Q.STATIC.

A two-tailed *p*-value of < 0.05 was considered to indicate statistical significance.

The ability of quantitative PET parameters (SUVmax and SUVmean) to differentiate between IPAS and pNETs was assessed using receiver operating characteristics (ROC) analysis. An area under the ROC curve (AUC) of 0.90–1 indicated excellent accuracy, 0.80 to < 0.90 good accuracy, 0.70 to < 0.80 fair accuracy, 0.60 to < 0.70 poor accuracy, and AUC 0.50–0.60 failed accuracy. ROC results were considered only if AUC was > 0.70^41^.

### Ethics approval

Our study was approved by the ethics committee of the University Hospital of Zurich and was conducted in compliance with ICH-GCP rules and the Declaration of Helsinki.

### Informed consent

Only patients with documented willingness to the use of their medical data for research and written informed consent for the scientific use of medical data were included.

## Results

Out of 498 cases, accessory spleens were detected in 63 PET/CT scans of 63 patients (12.2%). Of these, PET list mode data was available in 38 scans (60.3%; mean age 56.4, range 33–83 years). A single accessory spleen was present in 33 scans (31 scans with one accessory spleen and 2 scans with one accessory spleen and one pNET), two accessory spleens were presents in 5 scans. The majority of accessory spleens (79.1%) were located perisplenic, particularly medial to the spleen (55.8%; splenic hilum, gastrosplenic ligament, splenorenal ligament), followed by an intrapancreatic location (16.3%; IPAS), and 4.6% were ectopic (splenic vessels and paracolic—Table 1).

**Table 1:** Demographic data of study subjects and morphological data of accessory spleens and pNETs (values are given as mean ± standard deviation and range) for the entire cohort of patients (43 accessory spleens, 7 IPAS and 9 pNET).

Demographic data of subjects			
BMI, kg/m^2^ (range)	26.8 ± 4.4 (20.1–36.6)		
Injected tracer activity, MBq (range)	132.7 ± 20.9 (81.4–164.4)		
Time per bed, s (range)	134.0 ± 21.7 (120.0–180.0)		
Scan time post injection, min (range)	60.4 ± 6.2 (52.0–79.0)		
Volume of the spleen, mL	174.9 ± 43.4 (81.0–296.0)		

Lesion	Number, n (%)	Maximal diameter (mm)—CT	Volume (mm^3^)—CT
Accessory spleen—total	43 (100%)	13.5 ± 4.7 (6–31)	1477.3 ± 1807.9 (151–11,200)
Perisplenic	34 (79%)	13.8 ± 5.2 (6–31)	1636.1 ± 2054.8 (151–11,200)
Superior	0	–	–
Lateral	0	–	–
Inferior	3 (6.9%)	16.7 ± 4.1 (12–20)	2173.4 ± 1684.3 (630–3970)
Posterior	2 (4.6%)	11.5 (11–12)	754.5 (720–789)
Anterior	5 (11.6%)	10.8 ± 5.0 (6–18)	851.8 ± 927.7 (151–2420)
Medial	24 (55.8%)	14.2 ± 5.4 (9—31)	1805.8 ± 2322.2 (333–11,200)
Ectopic	9 (21%)	11.4 ± 3.8 (8–17)	877.2 ± 207.7 (367—1120)
Intrapancreatic	7 (16.3%)	12.3 ± 0.5 (12–13)	932.6 ± 89.9 (859–1120)
Splenic vessels	1 (2.3%)	17.0	1000.0
Colon wall	1 (2.3%)	8.0	367.0
pNET—total	9 (100%)	18.5 ± 3.9 (12–24)	2030.8 ± 977.7 (654–3540)

**Figure 3:**
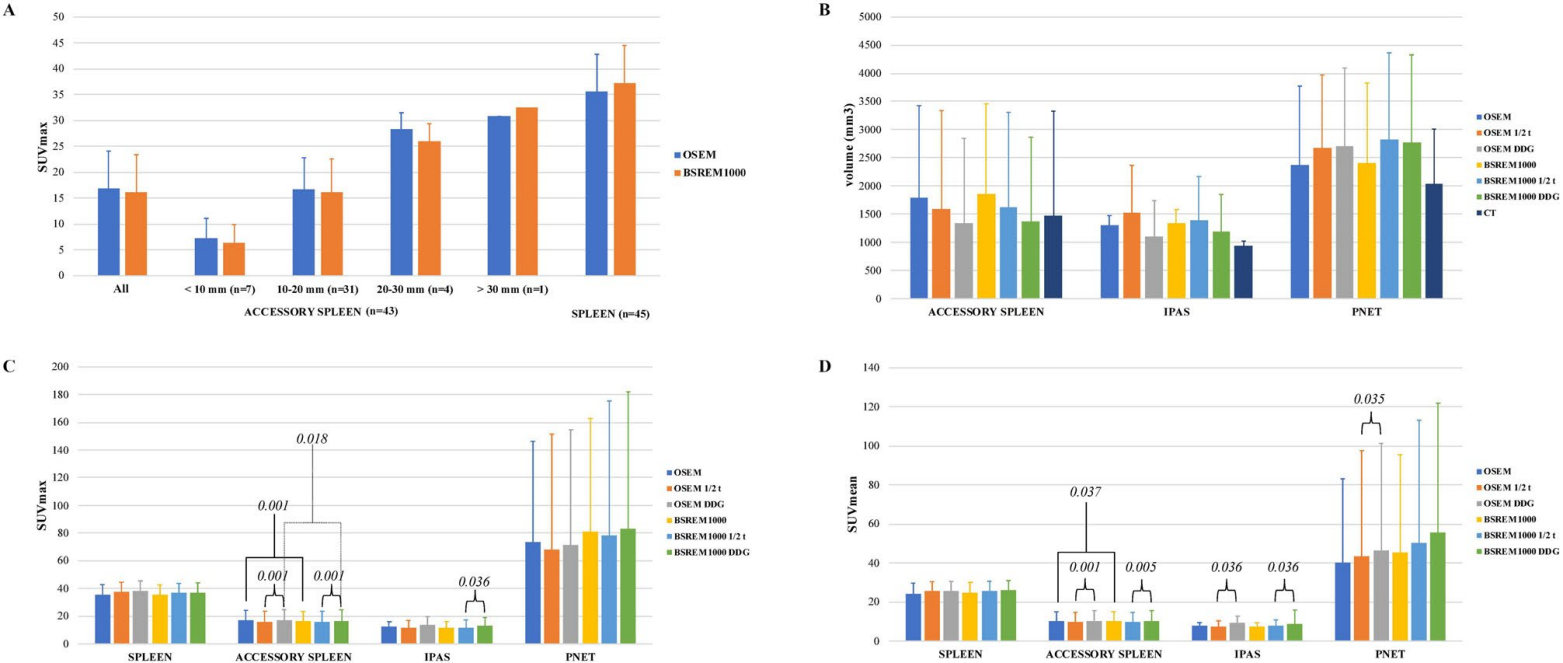
SUVmax of accessory spleens according to size (CT diameter of the lesions, mm) and spleen measured on OSEM and BSREM_1000_ reconstruction (**a**). PET volume (mm^3^) and CT volume (**b**), SUVmax (**c**) and SUVmean (**d**) of spleen, accessory spleen, IPAS and pNET among different PET reconstructions in the subcohort of exams acquired retrospectively (42 accessory spleens, 6 IPAS and 8 pNET). Significant *P*-values of Wilcoxon signed-ranks test, performed for accessory spleens, IPAS and pNET among different reconstructions, are reported in the SUVmax (**c**) and SUVmean panel (**d**). Note, for readability, only selected significant p-values of relevant comparisons are shown, which support the results reported in the text.

^68^Ga-DOTATATE uptake and size of accessory spleens were correlated, SUVmax of accessory spleens according to size measured on OSEM and BSREM_1000_ reconstruction is shown in Fig. 3a.

Significant correlations were observed between diameter (mm) and SUVmax of accessory spleens:

- in OSEM reconstruction with r^2^ = 0.779, *p* = 0.001, 95% CI [0.515, 0.853] for Pearson correlation, and a coefficient of 1.266, with standard error equal to 0.167, *p* = 0.001 and r^2^ = 57.21% for the linear regression equation;
- in BSREM reconstruction with r^2^ = 0.725, *p* = 0.001, 95% CI [0.598, 0.885] for Pearson correlation, and a coefficient of 1.246, with standard error equal to 0.178, *p* = 0.001 and r^2^ = 53.43% for the linear regression equation.

Out of 498 cases, pNET lesions (primary and metastatic) were detected in 9 PET/CT scans (1.8%; mean age 64.2, range 44–74 years), 2 with 1 accessory spleen and 1 pNET already included in the accessory spleen cohort.

Regarding the sub-cohort of patients retrospectively acquired and analyzed for the DDG reconstructions, results of lesion quantitation (42 accessory spleens, 6 IPAS and 8 pNET) with and without DDG (*R* > 10.0) are shown in Fig. 3b–d. In all reconstructions, SUVmax and SUVmean were significantly lower in accessory spleens compared to the spleen. *P*-values of Mann–Whitney U-test of PET/CT parameters (SUVmax and SUVmean) among the three samples (all accessory spleens, IPAS and pNETs) in each reconstruction are shown in Fig. 3c,d. *P*-values of Mann–Whitney U-test of PET/CT parameters (SUVmax and SUVmean) among all lesions (all accessory spleens, IPAS and pNETs together) in each reconstruction are given in Table 2.

**Table 2:** *P*-values of Mann–Whitney U-test of PET/CT parameters comparing the three samples (all accessory spleens, IPAS and pNETs) in each reconstruction in the subcohort of exams acquired retrospectively (42 accessory spleens, 6 IPAS and 8 pNET), as well as *P*-values of Wilcoxon signed-ranks test of PET/CT parameters of all lesions (42 accessory spleens, 6 IPAS and 8 pNET) among different reconstructions in the subcohort of exams acquired retrospectively.

	PET/CT parameters	BSREM_1000_ vs. OSEM	BSREM_1000_ (DDG 10.0) vs. OSEM (DDG 10.0)	OSEM (DDG 10.0) vs. OSEM (1/2)	BSREM_1000_ (DDG 10.0) vs. BSREM_1000_ (1/2)
All lesion* (accessory spleen, IPAS and pNET)	SUVmax	0.010	0.126	0.001	0.001
	SUVmean	0.297	0.482	0.001	0.001
	PET volume	0.136	0.152	0.001	0.086

Reconstructions**	PET parameters	Intrapancreatic spleen vs all accessory spleen	Intrapancreatic spleen vs pNET	pNET vs all accessory spleen	
OSEM	SUVmax	0.166	0.057	0.024	
	SUVmean	0.121	0.044	0.029	
BSREM_1000_	SUVmax	0.117	0.034	0.014	
	SUVmean	0.114	0.044	0.018	
OSEM DDG 10.0	SUVmax	0.559	0.033	0.002	
	SUVmean	0.473	0.024	0.003	
BSREM_1000_ DDG 10.0	SUVmax	0.854	0.033	0.002	
	SUVmean	0.676	0.033	0.003	

All lesion*: p-value was calculated with Wilcoxon signed-ranks test.

Reconstructions**: p-value was calculated with Mann–Whitney U-test.

For the entire sub-cohort, SUVmax and SUVmean were able to distinguish pNET both from all accessory spleens and from IPAS, both in OSEM and in BSREM reconstructions, with the only exception of SUVmax of IPAS vs. pNET in OSEM. In particular, SUVmax can distinguish:

- Accessory spleens vs. pNET in OSEM (*p* = 0.024) and BSREM (*p* = 0.014);
- IPAS vs. pNET in BSREM (*p* = 0.034).

An even higher level of significance was achieved with DDG (*R* > 10.0), where SUVmax can distinguish:

- Accessory spleens vs. pNET in both OSEM-DDG and BSREM-DDG (*p* = 0.002 each);
- IPAS vs. pNET in both OSEM-DDG and BSREM-DDG (*p* = 0.033 each).

**Figure 4:**
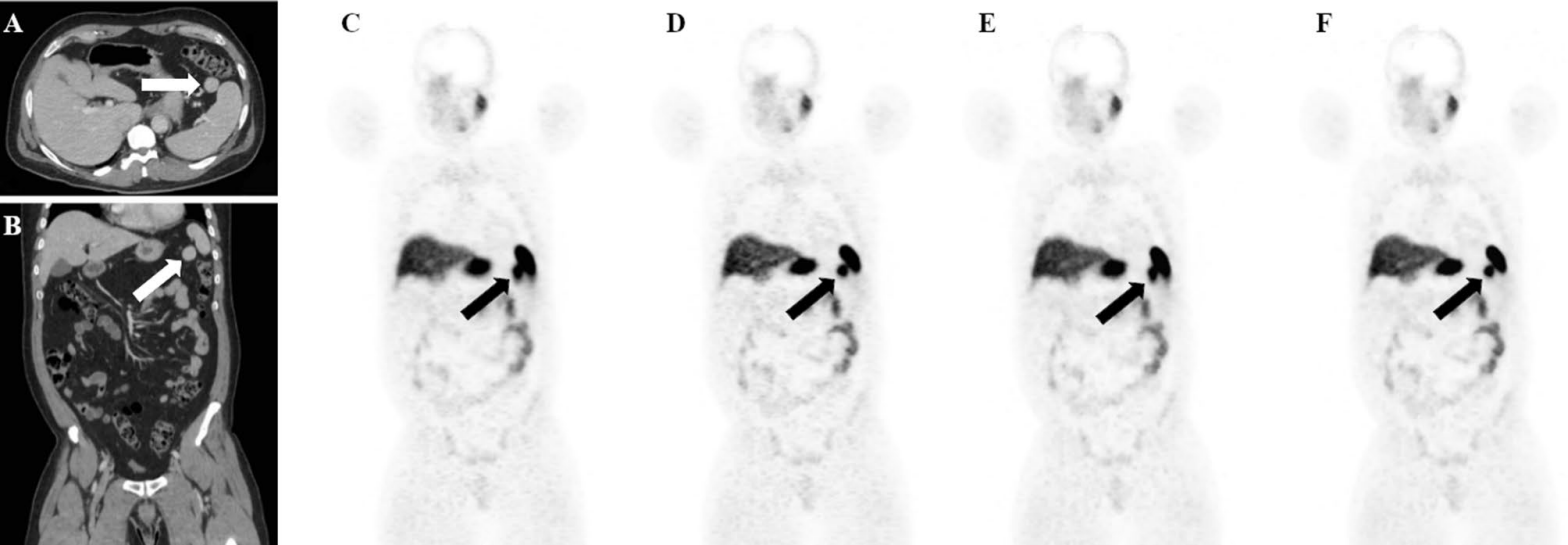
^68^Ga-DOTATATE PET/CT of a patient with an incidental accessory spleen, visible on contrast-enhanced CT images (**a**,**b**). The accessory spleen is better defined with DDG and yields higher SUVmax (30.8 on OSEM (**c**), 35.1 on OSEM-DDG (**d**), 32.4 on BSREM (**e**) 37.0 on BSREM-DDG (**f**)) and lower PET volume (10,750 mm^3^ on OSEM, 9320 mm^3^ on OSEM-DDG, 10,150 mm^3^ on BSREM 9130 mm^3^ on BSREM-DDG). Furthermore, BSREM-DDG shows better noise characteristics compared to OSEM-DDG.

Table 2 shows *p*-values of PET/CT parameters tested against each other in different reconstructions. Wilcoxon signed ranks test showed a significant difference of the following parameters:

- BSREM vs. OSEM: SUVmax (*p* = 0.001) and SUVmean (*p* = 0.037) of accessory spleens and SUVmax (*p* = 0.010) of all lesions were higher on BSREM;
- OSEM-DDG (*R* > 10.0) vs. OSEM ½: SUVmax and SUVmean of both accessory spleens and all lesions were higher on OSEM-DDG, while PET volume of both accessory spleens and all lesions (Fig. 4) was smaller on OSEM-DDG (all *p*-values < 0.001);
- BSREM-DDG vs. BSREM ½: SUVmax and SUVmean were higher for accessory spleens (*p*-values < 0.001 and 0.005, respectively), IPAS (both *p*-values < 0.036) and all lesions (both *p*-values < 0.001) on BSREM-DDG;
- BSREM-DDG vs. OSEM-DDG (*R* > 10.0): SUVmax of accessory spleens (p < 0.018) and SUVmean of pNET (p < 0.042) were higher on BSREM-DDG.

**Figure 5:**
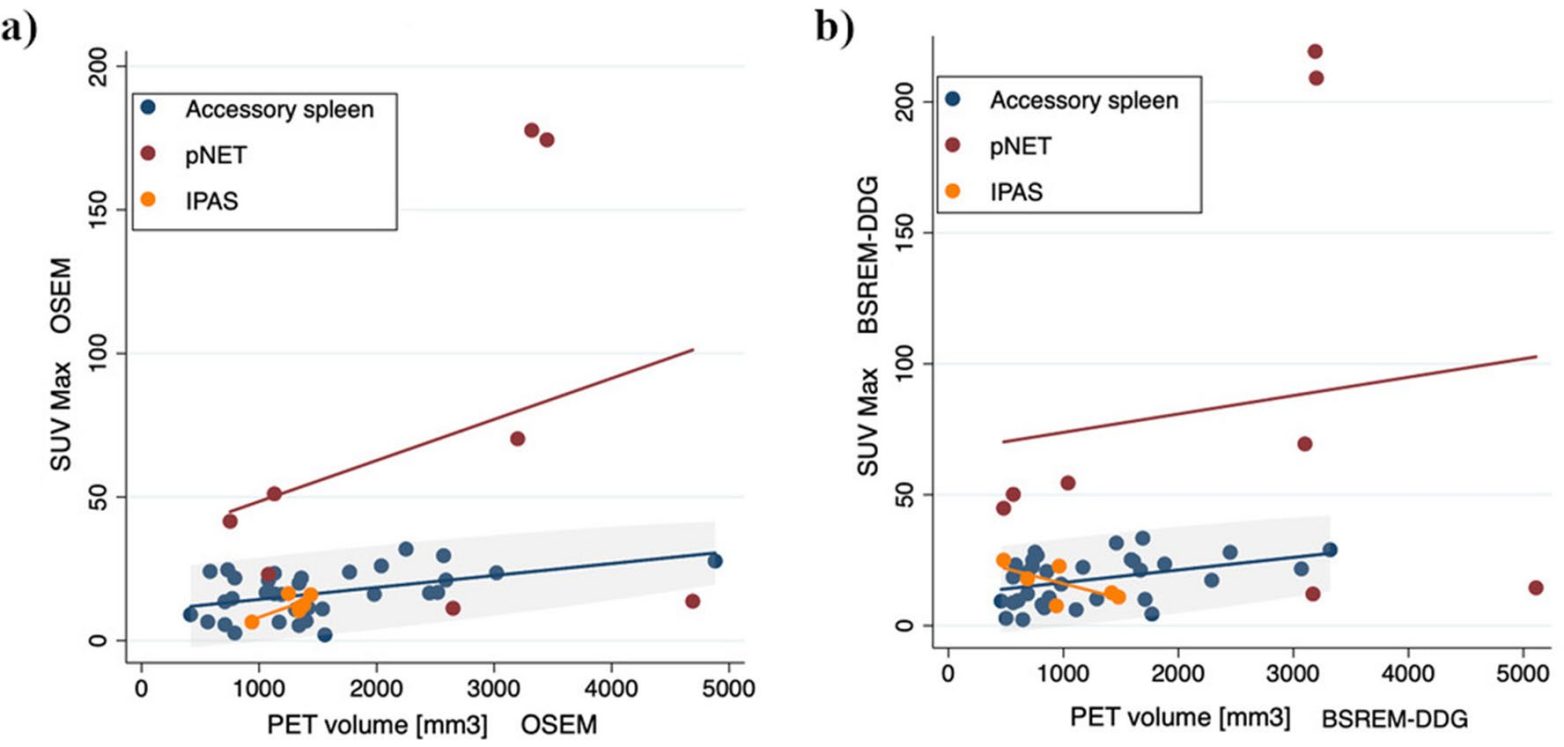
Scatter plots showing the relationship between SUVmax and PET volume of accessory spleens, IPAS and pNETs in OSEM (**a**) and BSREM-DDG (**b**), respectively, in the sub-cohort of patients retrospectively acquired (42 accessory spleens, 6 IPAS and 8 pNET).

The relationship between SUVmax and PET volume for accessory spleen, IPAS and pNET separately in each reconstruction (OSEM, OSEM-DDG, BSREM and BSREM-DDG) is shown in Fig. 5.

**Figure 6:**
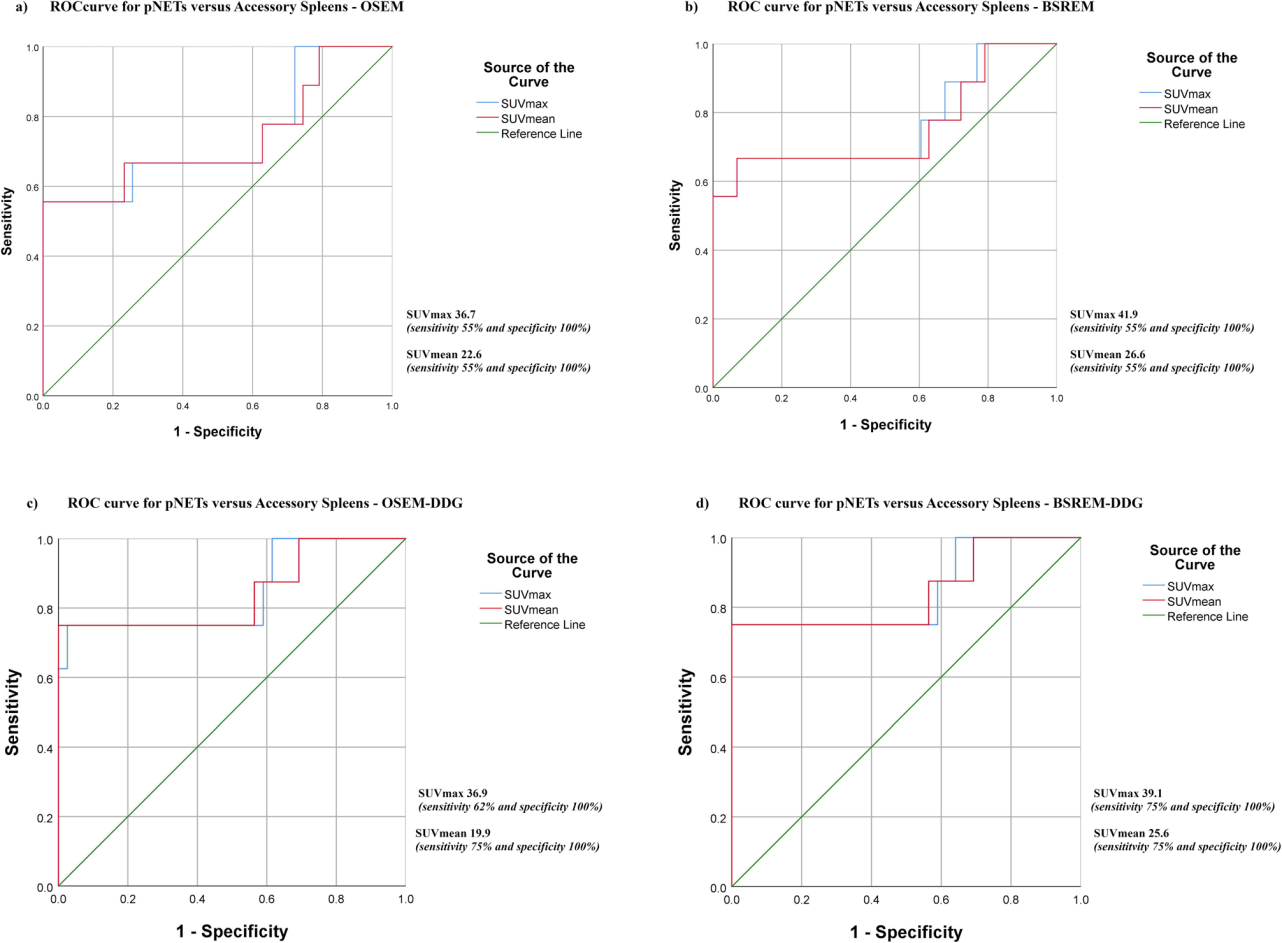
ROC curve of SUVmax and SUVmean for discriminating pNET and accessory spleens with OSEM (AUC = 0.742 and *p* = 0.024 of SUVmax; AUC = 0.734 and *p* = 0.029 of SUVmean (**a**)), BSREM (AUC = 0.765 and *p* = 0.013 of SUVmax; AUC = 0.755 and *p* = 0.017 of SUVmean (**b**)), OSEM-DDG (AUC = 0.846 and *p* = 0.002 of SUVmax; AUC = 0.843 and *p* = 0.002 of SUVmean (**c**)) and BSREM-DDG (AUC = 0.846 and *p* = 0.002 of SUVmax; AUC = 0.843 and *p* = 0.002 of SUVmean (**d**)) reconstructions in the sub-cohort of patients retrospectively acquired (42 accessory spleens, 6 IPAS and 8 pNET).

The ability of quantitative PET parameters of ^68^Ga-DOTATATE PET/CT to differentiate pNET and IPAS as well as accessory spleens was assessed using a ROC analysis with and without DDG (Fig. 6).

NET are a heterogeneous group of tumors and the uptake of ^68^Ga-labeled somatostatin analogue is higher in well-differentiated NET compared to poorly-differentiated NET, and correlates strongly with SSTR_2A_ receptor expression^42,43^. As shown in Fig. 3a, uptake of accessory spleens was rather homogeneous and was associated with the size of these organs. For this reason, we aimed to identify an SUVmax cut-off in each reconstruction to discriminate pNET and IPAS with a specificity of 100%, in order to avoid false positives. ROC results were considered only if AUC was > 0.70. OSEM-DDG and BSREM-DDG yielded good AUC (all > 0.84), while OSEM and BSREM yielded at least fair AUC (all > 0.73).

Cut-offs of SUVmax that discriminate pNET and IPAS with 100% specificity were as follows:

- OSEM: **SUVmax 19.7** (sensitivity 67%);
- BSREM: **SUVmax 22.5** (sensitivity 67%);
- OSEM-DDG (*R* > 10.0): **SUVmax 28.0** (sensitivity 75%);
- BSREM-DDG (*R* > 10.0): **SUVmax 25.0** (sensitivity 75%);

Cut-offs of SUVmax that discriminate pNET and accessory spleens with 100% specificity were as follows:

- OSEM: **SUVmax 36.7** (sensitivity 55%);
- BSREM: **SUVmax 41.9** (sensitivity 55%);
- OSEM-DDG (*R* > 10.0): **SUVmax 36.9** (sensitivity 62%);
- BSREM-DDG (*R* > 10.0): **SUVmax 39.1** (sensitivity 75%).

## Discussion

Our study sought to investigate whether quantitative PET parameters of ^68^Ga-DOTATATE-PET/CT can differentiate intrapancreatic accessory spleens and pancreatic neuroendocrine tumors.

The major findings of our study are as follows: (1) both SUVmax and SUVmean are able to distinguish pNET from accessory spleens and IPAS, (2) SUVmax on the BSREM-DDG reconstruction yields the best results (p-value ≤ 0.002 for pNET vs. accessory spleens and ≤ 0.033 for pNET vs. IPAS), (3) different SUVmax cut-off between pNET and accessory spleens/IPAS were found for each reconstruction, (4) and BSREM-DDG reconstruction achieved the best ROC curve result [an SUVmax cut-off > 41.7 identifies a pNET with a specificity of 100% and a sensitivity of 75% (AUC 0.840)], (5) SUVmax of accessory spleens is linearily correlated with their volume.

^68^Ga-labeled somatostatin analogue PET/CT is the mainstay for the evaluation of the somatostatin receptor (SSTR) status of neuroendocrine tumors. Our results suggest a possible new indication for the use of ^68^Ga-DOTA-peptide PET/CT in clinical routine, confirming the ability of ^68^Ga-DOTATATE PET/CT to distinguish IPAS and pNET. This finding may obviate additional imaging studies (as Tc-99m-labeled colloids or Tc-99m-HDRBC) in some cases and prevent unnecessary biopsy and/or surgery in cases of intrapancreatic accessory spleens.

Moreover, the impact of reconstruction algorithms is one of the most relevant factors on the use of absolute quantitative methods for PET/CT. In a recent phantom study, Lantos et al. find that BSREM can outperform OSEM in terms of contrast recovery and organ uniformity for several PET tracers, included 18F-FDG and ^68^Ga-DOTATATE^44^. Therefore, we evaluated both reconstruction algorithms (OSEM and BSREM with a default β-value of 1000, selected based on previous studies) and assessed their impact on quantification.

Results of our study are in line with previously described findings, particularly with the phantom study by Lantos et al.^44^. In our study, Bayesian penalized reconstruction data yielded slightly better results than OSEM distinguishing pNET from IPAS by SUVmax (*p-*value ≤ 0.034 for BSREM vs. ≤ 0.057 for OSEM) and for distinguishing pNET from accessory spleens by SUVmax (*p-*value ≤ 0.014 for BSREM vs. ≤ 0.024 for OSEM). This finding could be explained with the fact that the majority of well differentiated pNETs are characterized by significantly higher somatostatin receptor expression compared to the physiological uptake in splenic tissue (ectopic or not). Previous studies on BSREM reconstruction on lung cancer^31,45,46^ showed that lesions with higher uptake tend to converge faster than lesions with lower activity. The same holds true for our study, where BSREM reconstruction lead to a greater difference between the SUVmax of pNET vs. accessory spleens and/or IPAS compared to OSEM.

Besides the image reconstruction algorithm, PET image quality may also be affected by respiratory motion, leading to reduced quantitative accuracy and seemingly increased tumor/lesion volume (Fig. 4). This problem affects particularly small lesions in the upper abdomen, such as accessory spleens, IPAS and pNET^47–50^.

In our study, DDG increased the ability of PET parameters (SUVmax and SUVmean) to discriminate pNET from IPAS (SUVmax *p-value* ≤ 0.033) and/or accessory spleens (SUVmax *p-value* ≤ 0.002) both in BSREM and OSEM reconstructions.

Furthermore, for all the lesions included in our study, DDG lead to a significant increase in SUVmax and SUVmean, both with BSREM and OSEM reconstructions (all *p-*values ≤ 0.001), while a significant decrease in PET volume was only found with OSEM reconstruction (*p-*value ≤ 0.001). These results are in line with a recent study of Catalano et al.^51^. They stated that motion correction reconstructions reduce the effect of image blurring that leads to an underestimation of radiotracer uptake and hence falsely low SUVmax and falsely increased PET volume. This finding is important, because BSREM is still not widely available, and because DDG reconstruction could be easily done off-line, also from raw data acquired on older systems. However, in other instances, where motion blurring might be the dominant blurring factor, DDG might be more useful for BSREM compared to OSEM.

These results outline the importance of using a motion correction method to optimize the outcome of PET imaging. Besides DDG, other methods allow for motion correction, with the most common methods typically relying on external devices for gating. However, their use is often hampered by a comparably complicated setup, by technical problems and by time constraints.

The impact of different reconstruction algorithms with or without DDG is highlighted by the ROC results. Our study has identified different SUVmax and SUVmean cut-off values for each reconstruction. Furthermore, the benefits of both BSREM reconstruction (more accurate lesion quantitation and reduced background noise) and motion-corrected data (reduced image blurring) yielded the best results in our study: The most accurate SUVmax cut-off value (SUVmax 39.1, AUC 0.846, sensitivity 75% and specificity 100%) to discriminate pNET vs. accessory spleens was achieved with BSREM-DDG.

Finally, our study reveals a positive linear correlation between SSTR2-positivity and the size of accessory spleens, with larger accessory spleens exhibiting higher SUVmax. Even if this result was expected due to the partial volume effect, the differences in SUVmax between different size of accessory spleens (diameter < 10 mm, 10–20 mm and > 20 mm respectively) highlighted in this study, has an important role to better distinguish IPAS from pNET, according to the dimension of the pancreatic lesion under evaluation.

In fact, IPAS are typically solid enhancing lesions of 1.1–2.5 cm in size, usually not exceeding 3 cm in size^7–9^. In our study, the maximal diameter of IPAS on CT was 12.3 ± 0.5 mm, while the maximal diameter of accessory spleens on CT was 13.5 ± 4.7 (6—31) mm (72% of accessory spleens were 10–20 mm in size). Therefore, the size difference between IPAS and accessory spleens might partly explain the higher accuracy of SUVmax cut-offs for discriminating pNET from accessory spleens compared to IPAS.

Although the SUVmax of larger IPAS may be in the range of the SUVmax of well differentiated pNETs, this may not be so relevant in clinical routine, since IPAS-specific radiological characteristics are easily identified in larger lesions (i.e. heterogeneous arterial contrast enhancement of the lesion at and same degree of venous contrast enhancement of the spleen^52^). On the other hand, the assessment of a pancreatic incidentaloma in the pancreatic tail is more challenging with smaller lesions. Moreover, in the last years the incidental discovery of pancreatic incidentalomas increased owing to technology advancement as outlined by Vagefi et al. (from 2002 to 2007, 60.4% vs. 40.3% in previous years; mean diameter 42 mm vs. 56 mm in previous years^53^).

In conclusion, an SUVmax > 42 identifies a lesion as pNET with a specificity of 100%, regardless of the reconstruction technique used.

Some limitations of our study are acknowledged. First, the comparably small sample size may have affected our results. Multicenter studies might contribute data from other PET/CT scanners, amplifying the sample size. However, careful scanner harmonization is a prerequisite for this purpose. Second, owing to the retrospective design and also owing to ethical considerations, there was no histological standard of reference for accessory spleens. Instead, morphological imaging characteristics, stability over years and absence of abdominal neuroendocrine tumor history was used, which is a reasonable approach in our opinion. However, histopathology served as standard of reference for all pNETs in our study. Our study used an R-value threshold of 10 for DDG, and a β-value of 1000 for BSREM. Therefore, results of our study are limited to these parameter settings, and other settings might yield slightly different results. However, the thrust of our finding (higher SUV with BSREM and with DDG) is expected to be preserved.

## Conclusion

An SUVmax > 42 identifies a pNET with a specificity of 100%, regardless of the reconstruction technique used. DDG-based motion correction increases the ability of PET/CT parameters to discriminate IPAS/accessory spleens from pNET. DDG-based motion correction is beneficial particularly for the assessment of small lesions that are subject to respiratory motion, also in the upper abdomen. BSREM_1000_ leads to a significant increase of SUV parameters compared to OSEM, while DDG leads to a significant increase of SUV parameters and reduced PET volume compared to reconstructions without DDG. Hence, SUV cut-off values need to be adapted to different reconstruction settings.

## Data Availability

The datasets used and/or analyzed during the current study are available from the corresponding author on reasonable request.

## Abbreviations

AUC: Area under the curve
BMI: Body mass index
BPL: Bayesian penalized likelihood
BSREM: Block sequential regularized expectation maximization
CT: Computed tomography
CECT: Contrast-enhanced computed tomography
DDG: Data-driven gating
IPAS: Intrapancreatic accessory spleen
MIP: Maximum intensity projection
MR: Magnetic resonance
NET: Neuroendocrine tumor
OSEM: Ordered subset expectation maximization
PCA: Principal component analysis
PET: Positron emission tomography
ROC: Receiver operating characteristic
SiPM: Silicon photomultiplier
SSTR: Somatostatin receptor
SUVmax: Maximum standardized uptake value
TOF: Time of flight
VOI: Volume of interest
